# Asymmetry of Motif Conservation Within Their Homotypic Pairs Distinguishes DNA-Binding Domains of Target Transcription Factors in ChIP-Seq Data

**DOI:** 10.3390/ijms26010386

**Published:** 2025-01-04

**Authors:** Victor G. Levitsky, Vladimir V. Raditsa, Anton V. Tsukanov, Aleksey M. Mukhin, Igor F. Zhimulev, Tatyana I. Merkulova

**Affiliations:** 1 Department of System Biology, Institute of Cytology and Genetics, Novosibirsk 630090, Russia; raditsavv@bionet.nsc.ru (V.V.R.); tsukanov@bionet.nsc.ru (A.V.T.); mukhin@bionet.nsc.ru (A.M.M.); merkulova@bionet.nsc.ru (T.I.M.); 2 Department of Natural Science, Novosibirsk State University, Novosibirsk 630090, Russia; 3 Institute of Molecular and Cellular Biology, Novosibirsk 630090, Russia; zhimulev@mcb.nsc.ru

**Keywords:** chromatin immunoprecipitation followed by sequencing, transcription factor binding site prediction, cooperative binding of transcription factors, conservation of motifs, classification of transcription factors, direct binding of transcription factors

## Abstract

Transcription factors (TFs) are the main regulators of eukaryotic gene expression. The cooperative binding of at least two TFs to genomic DNA is a major mechanism of transcription regulation. Massive analysis of the co-occurrence of overrepresented pairs of motifs for different target TFs studied in ChIP-seq experiments can clarify the mechanisms of TF cooperation. We categorized the target TFs from *M. musculus* ChIP-seq and *A. thaliana* ChIP-seq/DAP-seq experiments according to the structure of their DNA-binding domains (DBDs) into classes. We studied homotypic pairs of motifs, using the same recognition model for each motif. Asymmetric and symmetric pairs consist of motifs of remote and close recognition scores. We found that asymmetric pairs of motifs predominate for all TF classes. TFs from the murine/plant ‘Basic helix–loop–helix (bHLH)’, ‘Basic leucine zipper (bZIP)’, and ‘Tryptophan cluster’ classes and murine ‘p53 domain’ and ‘Rel homology region’ classes showed the highest enrichment of asymmetric homotypic pairs of motifs. Pioneer TFs, despite their DBD types, have a higher significance of asymmetry within homotypic pairs of motifs compared to other TFs. Asymmetry within homotypic CEs is a promising new feature decrypting the mechanisms of gene transcription regulation.

## 1. Introduction

Transcription factors (TFs) are crucial proteins with sequence-specific DNA-binding activity, allowing them to regulate the transcription of target genes. TF binding sites (TFBSs) are regulatory elements with functional TF binding activity in genomic DNA. A motif is a general pattern of nucleotide context specificity that reflects this activity for a particular TF. Currently, the most popular genome-wide approach to deduce the motifs for a specific tissue/cell line/stage in vivo is the ChIP-seq experiment, which is based on chromatin immunoprecipitation (ChIP) and yields hundreds of binding loci (or peaks) for a given target TF [1,2]. Although this breakthrough approach has been widely used for more than 15 years [3], it remains expensive and technically challenging for many TFs. DNA affinity purification sequencing (DAP-seq) [4,5] is a recently developed alternative in vitro technique for genome-wide TFBS mapping.

A stable framework for the systematic massive analysis of TFBSs is the expandable hierarchical classification of TFs according to the structure of their DNA-binding domains (DBDs) (TFClass) [6,7,8,9]. This has been applied for mammals [7,8,9] and other eukaryotic taxa [10], including plants (Plant-TFClass) [11]. The first level of the hierarchy includes nine TF superclasses. They describe the general topology of DBDs. The second level, TF classes, implies structural and sequence similarities in the DBDs of TFs. The third level, TF families, relies on sequence similarities in the DBDs of TFs. For many lineages of eukaryotes, structurally similar TFs are very conservative [12]. Thus, no new superclasses have been identified in plant TFs compared to those found previously in mammals, and numerous TF classes are common to plants and mammals [11]. Here and below, we use the numeric notations from the TFClass database [6,7,8,9]. For instance, the superclass ‘Basic domain {1}’ contains the class ‘Basic leucine zipper factors (bZIP) {1.1}’.

In eukaryotes, TFs generally function as part of multiprotein complexes. The tissue- and stage-specific regulation of gene transcription is largely achieved through inherently combinatorial binding and the functions of multiple TFs [13,14,15]. As ChIP-seq technology does not separate direct and indirect interactions of target TFs in genomic DNA, several enriched motifs belonging to either the target TF or its collaborative (non-target) TFs are detected in each dataset of peaks. Below, these motifs are referred to as Anchor and Partner, correspondingly. The conservation of a motif refers to its degree of similarity to the known binding sites for a respective TF. In terms of the motif’s recognition model, a higher recognition score implies a higher conservation of the motif. Comparisons of the conservation of the Anchor and various Partner motifs are critical to explain the binding in vivo specificity of a target TF [15,16]. The analysis of co-occurring motifs in ChIP-seq data can indicate possible cooperation between target and partner TFs and suggest structural variability in the motifs of target TFs. For example, the propensity to interact with closed chromatin is a particular feature of a special group of TFs, the pioneer TF [17,18,19,20].

**Figure 1:**
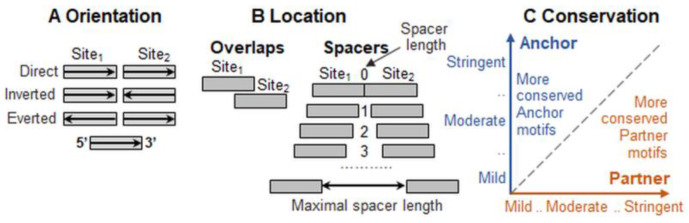
Main principles of CE classification. (**A**) Mutual orientation of motifs, (**B**) their overlaps or spacers, and (**C**) ratio of their conservation. The latter was defined only for heterotypic CEs [21]. Blue/red colors mark Anchor/Partner motifs. Axes X/Y imply the stringency of the recognition thresholds for the Partner/Anchor motifs.

To unravel the structure of a TFBS motif’s grammar below the first hierarchical level of individual motifs, the term composite element (CE) [21] is used as the simplest unit of the second level. A CE is a pair of motifs of two TFs whose co-occurrence close to each other in genomic DNA is observed more frequently than expected by chance. Several main attributes of CEs are generally considered [21,22,23,24,25,26]. First, homotypic and heterotypic CEs imply pairs of motifs of identical or distinct recognition models. Second, the different orientations of motifs include the head-to-tail case (Direct), where both motifs are in the same DNA strand, and the tail-to-tail (Inverted) and head-to-head (Everted) cases, which denote the opposite strands of two motifs (Figure 1A). Third, there are CEs with overlaps of motifs and with spacers between them (Figure 1B). For heterotypic CEs, we proposed to draw a scatterplot of the conservation of motifs of Anchor and Partner TFs, thereby detecting the trends towards greater conservation of one of these two motifs (Figure 1C). Hence, the fourth attribute is the presence or absence of systematic difference in the conservation of two participant motifs within pairs, i.e., all CEs are divided into asymmetric and symmetric.

Numerous large-scale experimental and theoretical analyses have indicated that besides the class of C2H2 zinc finger factors {2.3} (C2H2 ZF), paralogous TFs from the same classes have very similar DNA sequence preferences [10,12,16,27,28,29,30]. TFs of various classes have different abilities to form multimers. TFs from the Basic leucine zipper factor (bZIP) {1.1} and Basic helix–loop–helix factor (bHLH) {1.2} classes do not function as monomers. E.g., the motif of bHLH TFs, E-box CANNTG, binds a dimer of TFs, i.e., homotypic [31] CEs of E-box motifs can imply a TF tetramer. However, TFs of the Nuclear receptors with C4 zinc fingers {2.1} class (NR) function either as monomers, dimers, or multimers of several units arranged with various overlaps or spacers of different lengths [32,33]. Thus, CEs in DNA can correspond to either TF dimers or multimers with a greater number of subunits.

We recently proposed the approach MCOT (Motif Co-Occurrence Tool) for CE prediction in ChIP-seq data [21,34,35]. Unlike other tools [22,23,24,25,26], it uses a single ChIP-seq dataset to detect both CEs with spacers and overlaps of motifs, and applies multiple recognition thresholds to predict each motif, thereby distinguishing between asymmetric and symmetric CEs. We have shown that regardless of the target TF of the ChIP-seq experiment, CEs with more conserved Partner motifs are substantially more abundant compared to those with more conserved Anchor motifs [34]. However, the asymmetry of motifs within homotypic CEs has not yet been proposed and investigated.

In this study, we propose an approach to estimate the asymmetry of motif conservation within homotypic CEs. To validate this approach, we performed a massive analysis of ChIP-seq and DAP-seq data to test whether there are differences among the target TFs of various classes in terms of the significance of the asymmetry of their motif conservation in homotypic CEs. To avoid issues with distinction between structurally variated homotypic CEs with an overlap of motifs, we focused only on CEs with a spacer. We showed that target TFs of all classes avoid the enrichment of symmetric homotypic CEs, while the enrichment of asymmetric CEs depends on the class of the target TF. We found the greatest significance of asymmetry within homotypic CEs for motifs of TFs from three classes: two classes common to mammals and plants, bHLH and bZIP, and the mammalian class of p53 domain factors {6.3}.

## 2. Results

### 2.1. Definition and Examples of Asymmetric CEs

We earlier proposed the MCOT software package for the detection of spaced and overlapped pairs of co-occurring motifs for a single dataset of ChIP-seq peaks [21,34,35]. The MCOT requires an input set of DNA sequences and a given motif of a target TF (Anchor) representing its DNA-binding specificity. Here, we develop an approach to reveal the asymmetry in the conservation of motifs within their co-occurring pairs represented by the same motif models, i.e., homotypic CEs. Therefore, the nucleotide context pattern of homotypic CEs can estimate the cooperative binding of a target TF. Since target TFs and structurally similar partner TFs can have similar DNA specificity, this pattern may also be due to the co-occurrence of motifs of two distinct TFs. Here, MCOT applies the traditional position weight matrix (PWM) model to recognize the motifs of target TFs.

**Figure 2:**
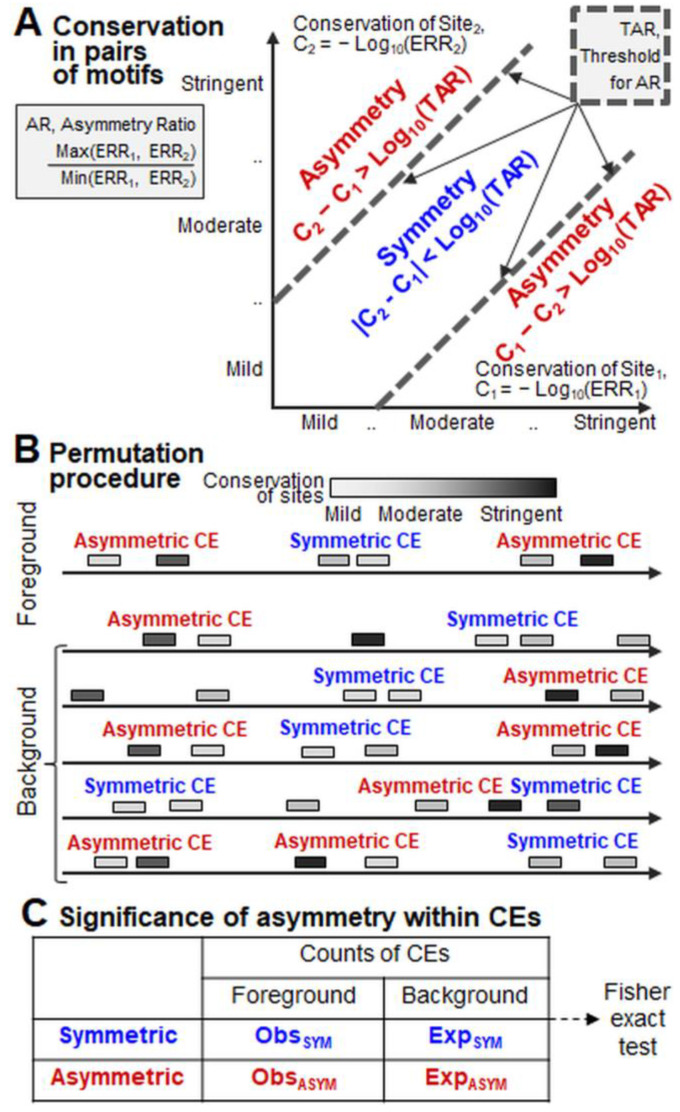
Detection of asymmetric and symmetric CEs. The blue/red fonts indicate symmetric and asymmetric CEs. (**A**) Distinguishing of asymmetric and symmetric CEs. The conservation of a site reflects its expected recognition rate (ERR) (see Section 4.3). The asymmetry ratio (AR) for a CE is the ratio of the conservation of its two sites, the largest to the smallest. (**B**) To detect CEs, the permutation [21] procedure prepares a background set of sequences. Here, ‘foreground’ means one peak and ‘background’ denotes its multiple versions with permuted sites (see Figure 2 in [21]). The numbers of asymmetric and symmetric CEs in the foreground and background sets are counted. (**C**) Fisher exact test for the 2 × 2 contingency table estimates the significance of asymmetry within a CE.

MCOT classifies homotypic CEs by the relative orientation and location of motifs, (Figure 1A,B). Here, we consider only CEs with spacers. CE annotation requires recognition profiles for both participating motifs. For each sequence, each profile lists predicted sites matching a motif. For each site, its start/end positions in a sequence, DNA strand, and conservation are indicated. For a given recognition score of a motif model, we define the conservation as the frequency of predicted sites with equal or larger recognition scores in the whole-genome set of promoters of protein coding genes, considering the common logarithm {−Log_10_(ERR)} of the expected recognition rate (ERR) (see Section 4.3). The difference between the conservation of two sites of the CE defines whether it is asymmetric or symmetric: |C_2_ − C_1_| = |−Log_10_(ERR_2_/ERR_1_)|. This is the uniform definition for heterotypic and homotypic CEs. Thus, the ratio of two ERR values, or the asymmetry ratio (AR), is a measure of asymmetry: AR = Max (ERR_1_, ERR_2_)/Min (ERR_1_, ERR_2_). The threshold for the AR (TAR) separates asymmetric and symmetric CEs. Two dashed lines corresponding to the TAR value (Figure 2A) divide the entire space of all pairs of conservation of two motifs (C_1_, C_2_) into distinct areas of points near and far from the diagonal. For example, a TAR value of 2 means that one ERR value twice exceeds the other, i.e., ERR_2_/ERR_1_ = 2 or ERR_1_/ERR_2_ = 2. Next, we count the total number of asymmetric and symmetric CEs in the foreground and background sets of sequences (Figure 2B). The foreground set consists of ChIP-seq peaks. For each peak, multiple background sequences are generated by the independent permutation of the sequential order of (1) minimal non-overlapping groups of predicted sites and (2) spacers between these groups [21]. Finally, the Fisher exact test assesses the significance of asymmetry in the conservation of motifs within CEs (Figure 2C).

### 2.2. Asymmetric CEs for the Target TF ARF5 in A. thaliana

**Figure 3:**
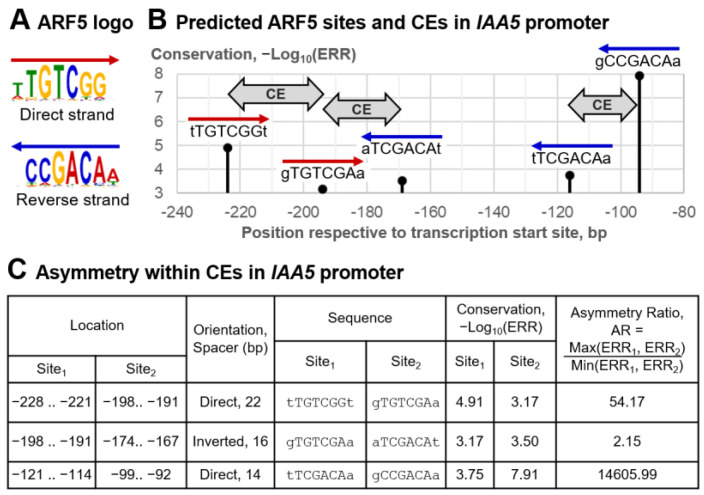
Asymmetric homotypic CEs of the ARF5 TF in the promoter of *A. thaliana* IAA5. (**A**) The ARF5 motif derived from DAP-seq [4,39] dataset for ARF5 TF. (**B**) Predicted five ARF5 BSs and three homotypic CEs in the promoter of IAA5 gene. (**C**) Summary of asymmetry of sites within three CEs: the first and third CEs have a strong asymmetry; the second CE has a moderate one. The last column shows the TAR value. CEs have a maximal spacer length of 30 bp and a maximal ERR of 0.001 (see Section 4.3).

In this section, we consider the target TF ARF5, a master regulator of auxin-dependent gene transcription in plants [36]. ARF5 belongs to the plant-specific TF class B3 {9.*} (superclass β-Barrel DNA-binding domains {9}); the asterisk here and below marks plant-specific clades in the Plant-TFClass [11], i.e., those not previously known in mammals [6,7,8,9]. The enrichment of the homotypic CEs of ARF5 motifs has been confirmed [4,37] and proven experimentally [38]. We used the DAP-seq dataset of ARF5 peaks [4,39] and performed a de novo motif search [40] to define the ARF5 motif (see Section 4, Section 4.1 and Section 4.2). Figure 3A depicts its logo. Next, we applied MCOT to this dataset with the following parameters: a maximal spacer length of 30 bp, ERR value of 0.001, and TAR value of 1.5 (see Section 4.3). We show homotypic asymmetric CEs for the AT1G15580 (*IAA5*) gene promoter as an example. The analysis of multiple transcriptomic datasets revealed this gene among the twenty top-ranked auxin-induced genes in *A. thaliana* [38]. Among the five predicted ARF5 sites in the promoter of the *IAA5* gene, two sites match the perfect ARF5 consensus (TGTCGG, Figure 3A). Five sites formed three distinct CEs (Figure 3B,C). The TAR value 1.5 marks two CEs as highly asymmetric (AR > 50), and the third CE has moderate asymmetry (AR > 2).

**Figure 4:**
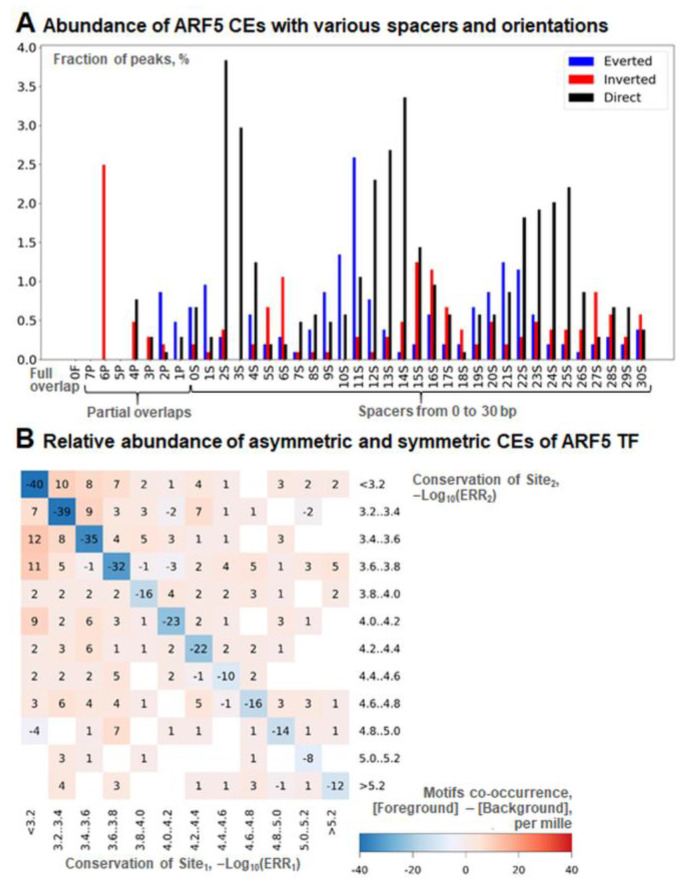
Structural heterogeneity of homotypic CEs for TF ARF5 according to DAP-seq data. (**A**) Abundance of CEs with various orientations, overlaps, and spacers. Blue, red, and black colors denote Everted, Inverted, and Direct orientations of motifs in pairs. The letters in the labels below the *x* axis from left to right mean full/partial overlaps of sites (‘F’/‘P’), and spacer (‘S’). The numbers preceding these letters denote, respectively, the distance between the nearest borders of two sites, the length of overlap, and the length of the spacer. Axis *y* shows the percentage of peaks containing CEs of a particular structure. (**B**) Relative abundance of asymmetric and symmetric CEs. Red/blue colors mark enrichment/depletion according the per mille measure (see Section 4.3). The diagonal and all other cells refer to symmetric and asymmetric CEs. Axes X/Y show ranges of the conservation of sites in CE, Site_1_, and Site_2_; indices 1 and 2 mean sites located closer to 5′ and 3′ ends of a DNA sequence.

Further, for the DAP-seq dataset of ARF5 peaks [4,39], we decided to test the abundance of homotypic asymmetric CEs of the ARF5 motif. We applied the web server MCOT [35] with the same parameters as described above. The distribution of homotypic CEs with respect to the mutual locations and orientations of sites (Figure 4A) follows the known pattern of the co-occurrence of ARF5 motifs [4,37]. The Direct repeats are enriched for the spacer lengths of 2–4, 12–15, and 22–25 bp. Adding the motif length (8 bp, Figure 4A) to these values yields distances of 10–12, 20–23, and 30–33 bp. These are multiples of the DNA double-helix turn length 10–11 bp. Peaks for Inverted and Everted orientations are found in the antiphase positions, as was observed earlier [4,37]. We found that these enriched CEs are asymmetric (Figure 4B). For any conservation of the ARF5 motif, the pairs of sites with almost equal conservation are depleted, while pairs of sites with distinct conservation are enriched (Figure 4B, blue cells on the diagonal compared to light brown cells beyond it). Overall, in the ARF5 peaks, the fraction of asymmetric CEs among all homotypic ones substantially exceeds that found for the background set (*p*-value < 3 × 10^−40^, Fisher exact test). Thus, the asymmetry of motif conservation is a specific feature of the homotypic CEs for the ARF5 TF.

### 2.3. Asymmetry Within CEs Depends on the Class of Target TF

The previous section confirmed the significant enrichment of asymmetric homotypic CEs for a particular target TF. It is tempting to test this phenomenon for target TFs from various classes. We compiled the benchmark collections of ChIP-seq and DAP-seq data (see Section 4.1). ChIP-seq data were extracted for *M. musculus* and *A. thaliana* from GTRD [41], and DAP-seq data for *A. thaliana* were taken from Plant Cistrome [4]. We performed the filtration of ChIP-seq and DAP-seq data. For ChIP-seq data, we ensured the significant enrichment [42] (*p* < 0.001) of known BS motifs of target TFs [10,28,29]. We performed a de novo motif search [40] and confirmed [43] that the deduced motifs were significantly similar to the known motifs of the target TFs (see Section 4.2). We categorized TFs according to their DBD into classes. Final benchmark collections of *M. musculus* ChIP-seq data and of *A. thaliana* ChIP-seq/DAP-seq data amounted to 1281 and 80/495 datasets, correspondingly (see Tables S1–S6).

**Figure 5:**
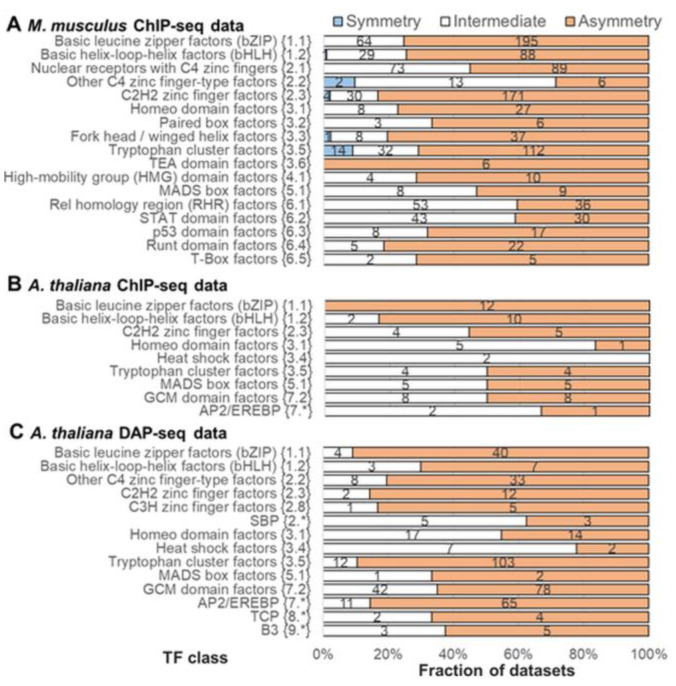
Abundances of homotypic asymmetric/symmetric CEs. (**A**) ChIP-seq data for *M. musculus*. (**B**,**C**) ChIP-seq/DAP-seq data for *A. thaliana*. Axis X shows the number of datasets. Blue/brown colors count datasets possessing a high significance of enrichment within homotypic symmetric/asymmetric CEs (*p*-value < 10^−10^). White color means that neither symmetric nor asymmetric CEs have high significance (*p*-value > 10^−10^). Axis Y displays TF classes from TFClass [7,8] (**A**) and Plant-TFClass [11] (**B**,**C**). For *M. musculus* ChIP-seq data and *A. thaliana* ChIP-seq/DAP-seq data, only classes with at least 6/2 and 3 datasets, correspondingly, are shown. Tables S7–S9 provide the significance for all TF classes and all benchmark collections. See also Figures S1 and S2. The asterisk (‘*’) marks plant-specific TF classes according the Plant-TFClass [11].

We applied MCOT to three benchmark collections to compare the abundance of significant homotypic symmetric and asymmetric CEs. We set [34] the threshold for the significance within homotypic asymmetric/symmetric CEs as *p*-value < 10^−10^ (Figure 2C). For each TF class in each collection, we identified the number of datasets with significant asymmetry or symmetry and categorized all others as ‘Intermediate’ (Figure 5). Here and below, the default TAR value of 1.5 is applied. Figures S1 and S2 show the results for TAR values of 1.1 and 2. For all collections and all TF classes, we detect a clear trend towards asymmetric CEs. However, the proportions of datasets following this trend are notably different for various classes. For the greatest collection of *M. musculus* ChIP-seq data, Figure 5A shows the 17 classes with the largest numbers of datasets. The six largest classes are bZIP, bHLH, NR, C2H2 ZF, Tryptophan cluster factors {3.5} (Tryp C), and Rel homology region (RHR) factors {6.1}. The bZIP and bHLH classes were found in over 75% of datasets with significant asymmetry. Only 1 dataset out of 118 datasets for the bHLH class showed significant symmetry. The C2H2 ZF and Tryp C classes showed fractions of 83% and 71% with significant asymmetry, each of them revealing a small fraction of significant symmetry (2% and 9%). The NR class was found in only 55% of datasets with significant asymmetry. Only 3 outlier classes out of 17, Other C4 zinc finger-type factors {2.2}, RHR, and STAT domain factors {6.2} (STAT), did not reach 50% of datasets for significant asymmetry (29, 40, and 41%, correspondingly). The ChIP-seq and DAP-seq data for *A. thaliana* (Figure 5B,C), in general, confirm the trends noted above for *M. musculus*. Namely, the bHLH and bZIP classes were found in high fractions of datasets with significant asymmetry within homotypic CEs (above 70%). We did not detect any datasets with significant symmetry in the *A. thaliana* data.

**Figure 6:**
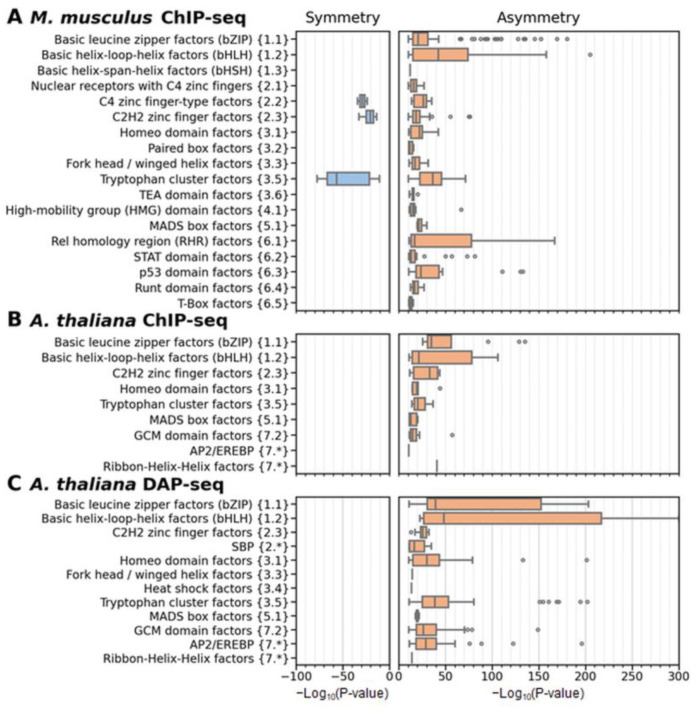
Distributions of the significance of enrichment of homotypic asymmetric/symmetric CEs as a function of the class target TF. (**A**) ChIP-seq data for *M. musculus.* (**B**,**C**) ChIP-seq/DAP-seq data for *A. thaliana.* Hierarchical classifications of target TFs by the structure of DBDs were derived from TFclass [7,8] and Plant-TFclass [11] (see Section 4.3). Axis X indicates the significance of enrichment by Fisher exact test with −Log_10_ (*p*-value), calculated by MCOT [21]. Brown/blue colors imply significant enrichment towards asymmetry/symmetry; for each TF class, we considered only datasets possessing enrichment towards asymmetry or symmetry according to the results from Figure 5. Axis Y shows TF classes. The boxplots depict the distributions of the Q_1_, Q_2_, and Q_3_ quartiles of the fractions of the datasets with certain values of significance (−Log_10_ (*p*-value)). Whiskers on either side of the Q_1_/Q_3_ respect the minimum/maximum values if they were located within 1.5 interquartile ranges (IQR = Q_3_ − Q_1_) from Q_1_/Q_3_; otherwise, they are equal to {Q_1_ − 1.5 × IQR}/{Q_3_ + 1.5 × IQR}, respectively. In the latter case, we marked all other points as outliers. The asterisk (‘*’) marks plant-specific TF classes according the Plant-TFClass [11].

To identify subtle differences in the significance of asymmetric homotypic CEs between target TFs of different classes, we computed the distributions of the significance of either asymmetry or symmetry within homotypic CEs for all collections and TF classes (Figure 6). These distributions strongly support the leading advantage of the bHLH and bZIP classes over all the remaining classes. The variation in the border between asymmetric and symmetric homotypic CEs (TAR values of 1.1 and 2) confirms this conclusion for all benchmark collections (Figures S1–S4, Tables S7–S9). bHLH and bZIP are large classes with the common highest ranks in the significance of asymmetry among all common mammal and plant TF classes in three benchmark collections. Besides them, only one large common class, Tryp C, can be noted. Two other classes specific to mammals leading in asymmetry, p53 domain factors {6.3} and RHR (Figure 5A and Figure 6A), can be noted. The collection of plant ChIP-seq data is too small, so according to the plant DAP-seq data, besides the bHLH and bZIP classes, the next highly significant asymmetry within the CEs is found for target TFs from the AP2/EREBP {7.*} and Tryp C classes (Figure 5C and Figure 6C).

Our results indicate that the DBD structure of TFs from different classes participates differently in the formation of multiprotein complexes regulating transcription. Consequently, this structure predetermines a different degree of flexibility of cooperative mechanisms for multiprotein complexes defined by target TFs of different classes. Thus, the interaction of TFs with sites of higher affinity promotes TF-DNA interactions with neighboring sites of lower affinity. We found that for target TFs from bZIP and bHLH classes, this cooperative binding is most pronounced.

### 2.4. CEs of Pioneer TFs Are More Asymmetric Compared to CEs of Other TFs

**Figure 7:**
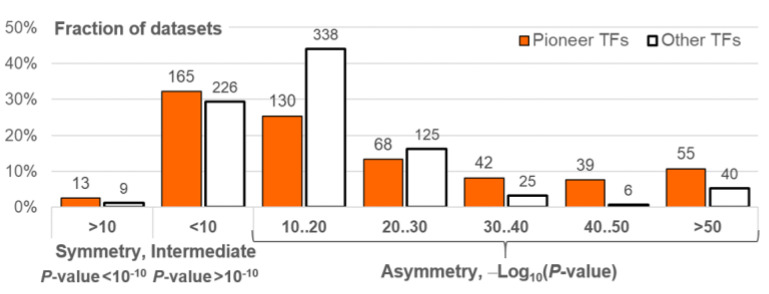
Distributions of the significance of enrichment of homotypic asymmetric CEs for ChIP-seq datasets of target TFs with and without proven pioneer activity. Target TFs from the benchmark collection for *M. musculus* ChIP-seq data were considered. Orange and white columns indicate TFs with and without proven pioneering activity. Axis *x* denotes the significance of enrichment. The groups Symmetry, Intermediate, Asymmetry 10‥20, Asymmetry 20‥30, etc., imply the significant enrichment of symmetric CEs (*p*-value < 10^−10^), a lack of high significance in either direction (*p* > 10^−10^), and the significant enrichment of asymmetric CEs (10^−20^ < *p*-value < 10^−10^, 10^−30^ < *p*-value < 10^−20^, etc.), respectively. Axis *y* means the fraction of datasets; labels above columns show the number of datasets.

The previous section demonstrated the general tendency of the asymmetry of motif conservation within homotypic CEs of target TFs from all classes. A specific group of eukaryotic TFs, pioneer TFs, are able to initiate the access of transcription machinery to the regulatory regions of genes in closed chromatin [19]. These TFs recruit other TFs and, therefore, they should have a more pronounced ability to interact directly with DNA than other TFs. Hence, we asked whether homotypic CEs of pioneer TFs had higher asymmetry compared to those of other TFs. We considered only the benchmark collection of ChIP-seq data for *M. musculus*, since pioneer TFs are substantially better known in mammals [19]. We compiled references to experimental evidence of pioneer activity for 63 human or murine TFs (Table S10). We compared the significance of asymmetry within homotypic CEs for TFs with proven pioneer activity and for other TFs (Figure 7). We identified datasets with a significant enrichment of symmetric homotypic CEs (*p*-value < 10^−10^), no apparent enrichment towards either symmetry or asymmetry (*p*-value > 10^−10^) and partitioned datasets with significance towards asymmetry into several groups, as follows: 10^−20^ < *p*-value < 10^−10^, 10^−30^ < *p*-value < 10^−20^, etc. Obviously, the Symmetry and Intermediate groups do not distinguish between pioneer and other TFs. However, the stringent thresholds of significance certainly provide enrichment for the homotypic CEs of pioneer TFs (e.g., for *p*-value < 10^−30^, Fisher exact test shows *p*-value < 4 × 10^−16^). We confirmed similar significance for TAR values of 1.1 and 2 (Figure S5). Next, we confirmed the significance of the difference between pioneer and other TFs separately for target TFs from the Basic domain {1} and Helix–turn–helix domain {3} superclasses (Figure S6, Fisher exact test for TAR value of 1.5; *p*-value < 2 × 10^−7^ and *p*-value < 10^−5^, respectively). The correction for multiple comparisons supports the significance of these two superclasses. The corrected threshold of the significance is about 0.05/4/4 ≈ 3.1 × 10^−3^ (Bonferroni’s correction). We consider four TF superclasses and four intervals of asymmetry significance (from 10^−10^ < *p*-value < 10^−20^ to *p*-value < 10^−50^, Figure 7).

**Figure 8:**
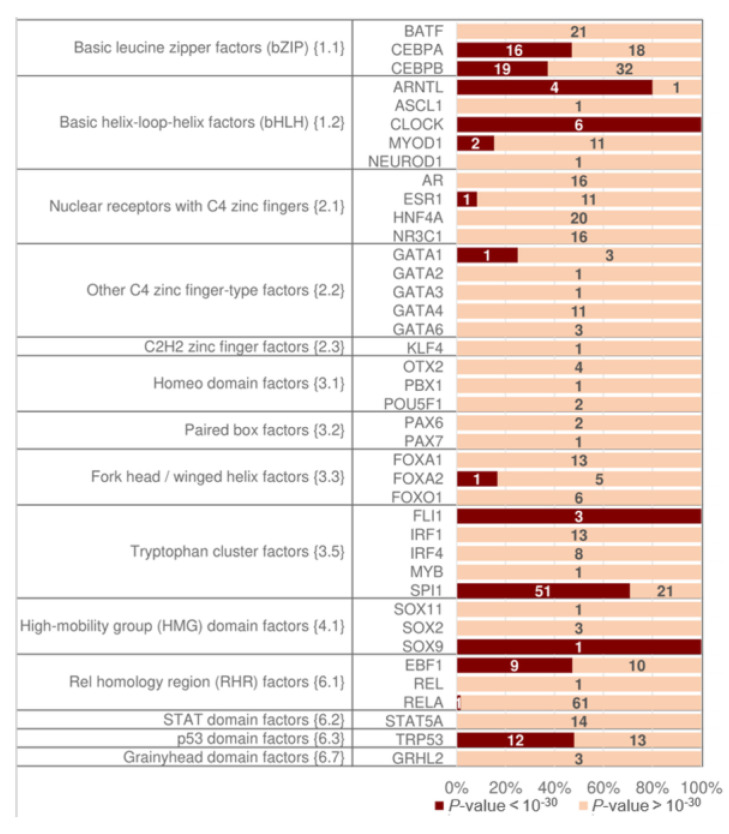
Pioneer target TFs with significant homotypic asymmetric CEs. Distributions of the significance of asymmetric CEs for datasets from the benchmark collection of *M. musculus* ChIP-seq data for the target TFs with and without proven pioneering activity. Axis *x* marks the number of ChIP-seq datasets. Axis *y* shows classes and names of TFs. The maroon and apricot stripes indicate the high significance of asymmetry within homotypic CEs (*p*-value < 10^−30^) and all remaining cases (*p*-value > 10^−30^). Table S10 lists TFs with proven pioneering activity used in analysis.

Finally, we tested pioneer TFs individually to determine which classes have higher asymmetry significance in homotypic CEs of pioneer TFs. Figure 8 shows the distribution of pioneer TFs from all classes. They are categorized by the presence/absence of highly significant asymmetry within homotypic CEs at a significance threshold of *p*-value < 10^−30^. Certain pioneer TFs from the bZIP and bHLH classes often have high significance; e.g., compare the TFs CLOCK and MYOD1 from the bHLH class. Pioneer TFs from other classes also reach high significance (TFs SPI1 and EBF1 from the classes Tryp C and RHR). The respective distributions for TAR values of 1.1 and 2 show similar trends (Figure S7).

### 2.5. Asymmetric Homotypic CEs Can Represent Pairs of Motifs of Distinct TFs

**Figure 9:**
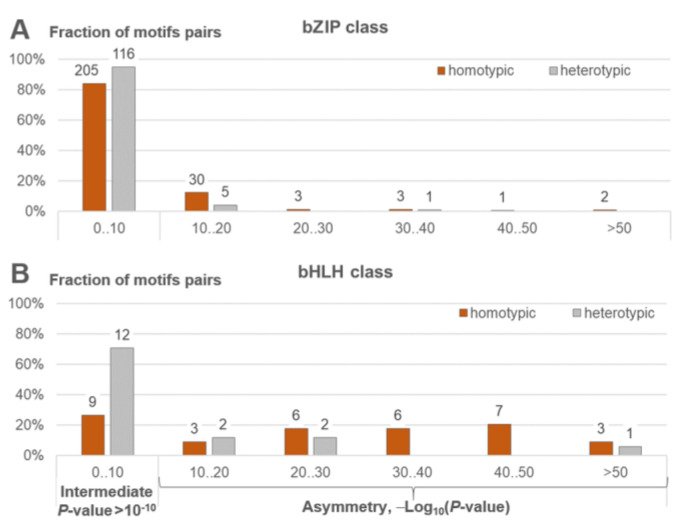
Distributions of the significance of enrichment of homotypic and heterotypic CEs for overlapping peaks in pairs of ChIP-seq datasets of distinct target TFs from the same TF classes. The homotypic and heterotypic CEs represent pairs of motifs for the same and distinct TFs (orange and grey columns, correspondingly). For a pair of ChIP-seq datasets respecting two distinct TFs (TF1 and TF2) from the same class, we considered one heterotypic (TF1-TF2) and two homotypic (TF1-TF1 and TF2-TF2) pairs of motifs. We considered CEs for target TFs from bZIP (**A**) and bHLH (**B**) classes. Axis *x* denotes the significance of enrichment. The groups Intermediate, Asymmetry 10‥20, Asymmetry 20‥30, etc., imply a lack of high significance in either direction (*p*-value > 10^−10^) and the significant enrichment of asymmetric CEs (10^−20^ < *p*-value < 10^−10^, 10^−30^ < *p*-value < 10^−20^, etc.), respectively. Axis *y* represents fractions of tested pairs of motifs; labels above columns show the number of pairs of motifs. For each ChIP-seq dataset, we ensured the enrichment of known motifs of target TFs (AME [42] and TomTom [43] tools, *p*-value < 0.001, Table S1). Two ChIP-seq experiments for two TFs have identical tissue/cell line or treatment conditions. Additionally, we required that (1) the overlap of peaks was significantly high [44], the number of overlapped peaks between two datasets was significantly high (above 50), and (2) that the TomTom tool [43] confirmed at least a weak similarity of enriched motifs with respect to the target TFs between two compared ChIP-seq datasets (see Section 4.4). See details in Supplementary Tables S11 and S12.

Now, we return to the hypothesis that the significant asymmetry within homotypic CEs can be due the co-occurrence of motifs of two structurally similar but distinct TFs from the same class. ChIP-seq data represent potential motifs of multiprotein complexes, which may include not only target TFs, but other TFs. Assigning two TFs to the same class determines similarities, yet small differences in their motif pairs may reproduce an asymmetric pattern of motif conservation in enriched pairs. Hence, in the analysis, we considered pairs of ChIP-seq datasets for target TFs from the same classes, ensuring the significance of the genomic overlap between their peaks (BEDtools) [44] and moderate similarity of motifs (*p*-value < 0.05) [43] in pairs of motifs respecting two distinct target TFs (see Section 4.3). According to the results above (Figure 5 and Figure 6), we included in this analysis only two classes of mammalian TFs, bZIP and bHLH. Among the most common large TF classes in eukaryotes [12], only these two classes possess TFs that act only as dimers [45]. We prepared pairs of ChIP-seq datasets annotated for the same tissue, cell line, or treatment conditions, targeting TFs from the same classes with a significant genomic overlap (see Tables S11 and S12). We applied MCOT to compare the significance of asymmetry within homotypic and heterotypic CEs. In particular, for a pair of TFs, TF1 and TF2, the homotypic and heterotypic CEs result in pairs of motifs: (TF1 vs. TF1, TF2 vs. TF2) and (TF1 vs. TF2), correspondingly. For both tested TF classes, we found that the enrichment of asymmetric CEs is notably higher for homotypic CEs than for heterotypic CEs (see Figure 9). According to Fisher’s exact test, the abundance of asymmetric CEs (*p*-value < 10^−10^) for homotypic CEs is significantly higher compared to those for heterotypic CEs (*p*-value < 3 × 10^−3^ (bZIP) and *p*-value < 6 × 10^−3^ (bHLH)). Consequently, motifs of structurally similar target TFs have a moderate impact on the detected enrichment of significant asymmetry within homotypic CEs. Nevertheless, co-occurring motifs belonging to the same TFs are a more universal rationale for this asymmetry, as the same results are found for all TF classes. Distant eukaryotic taxa and DAP-seq data exclude the action of any partner TFs. This conclusion supports the main results of our study.

## 3. Discussion

The prediction of TFBS motifs is an important step in deciphering the nucleotide context responsible for transcription regulation. TFs as main regulators of gene transcription have a basic cooperative action mode. To understand the molecular mechanisms of transcription, it is necessary to determine the relationships of individual TFBS motifs and to find CEs as co-occurring pairs of motifs. Notably, these pairs are overrepresented in functional regions of genomic DNA compared to random expectation. Since the beginning of the next-generation sequencing era, and, in particular, since the massive application of ChIP-seq technology, many tools have been proposed and consistently applied for CE prediction [22,23,24,25,26]. To support these studies, we have continuously developed our approach, MCOT [21,34,35]. So far, no CE prediction tool besides MCOT has been able to detect significant asymmetry or symmetry, or a lack of both within CEs. However, in previous studies [21,34], we analyzed the asymmetry of motif conservation only for heterotypic CEs (Figure 1C). In this study, we extended the definition of asymmetry to homotypic CEs (Figure 2A). This allowed a massive analysis of ChIP-seq and DAP-seq data and, in addition, revealed clear differences in the structure of homotypic CEs for target TFs from various classes.

To start our analysis, we chose the TF ARF5, a well-known master regulator of plant growth and development [46], and its known target gene *IAA5* [47], detected previously as a top-ranked [38] auxin-induced gene. TF ARF5 activates genes regulated by the plant hormone auxin. The *IAA5* gene belongs to the gene family Aux/IAA of transcriptional repressors. At low concentrations of auxin, ARF5 occupies its BSs in the promoters of auxin-responsive genes, but the direct interaction of Aux/IAA repressors with ARF5 inhibits the activity of ARF5. At a high concentration of auxin, this direct interaction is disrupted, and ARF5 becomes functional. As this process in plants stands at the core of the gene network, it has to be fine-tuned reliably. Three asymmetric homotypic CEs of ARF5 BSs in the promoter of the *IAA5* gene support the effective regulation of the *IAA5* gene under the action of ARF5 (Figure 3B,C). Next, we tested the enrichment of asymmetric homotypic CEs for the entire dataset of DAP-seq peaks [4,39] for the ARF5 TF. Although the specific pattern of the spacer length distribution in homotypic CEs for ARF5 (Figure 4A) was noted previously [4,37], the asymmetry of motif conservation within these homotypic CEs has not yet been identified (Figure 4B). The ARF5 TF belongs to the class B3 {9.*} (Table S3); the other seven members of this class have a substantially less significant enrichment of asymmetric homotypic CEs (see Table S9 for DAP-seq data—*p*-value < 2 × 10^−42^ for ARF5; the median for all other TFs from class B3 only slightly exceeds the threshold value of *p*-value < 10^−10^). These results motivated the analysis of the benchmark collections of ChIP-seq and DAP-seq data. Our main goal was to identify the relationship between the significance of asymmetry in homotypic CEs, on the one hand, and the structure and functions of target TFs on the other hand.

Remarkably, the results of the massive analysis of the abundance and enrichment of asymmetric and symmetric homotypic CEs (Figure 5 and Figure 6) clearly indicate that TFs from various classes have specific patterns in asymmetric homotypic CEs. Significant symmetric CEs (*p*-value < 10^−10^) are substantially depleted compared to asymmetric CEs for all TF classes. We detected them only in a few ChIP-seq datasets for *M. musculus* (Figure 5A and Figures S1A and S3A). These conclusions are quite expected; most likely, they reflect the widespread cooperative mechanism of action of eukaryotic TFs. However, the fractions of datasets with significant asymmetry (*p*-value < 10^−10^) clearly differ for target TFs from various classes. This is not so expected, and even slightly striking. Nevertheless, many earlier studies have indicated that this result is quite reasonable.

A possible stereochemical structure underlying both TF-TF and TF-DNA interactions defines the diversity of CE structures. For example, TFs of the bZIP class function only as dimers; two half-sites form the binding site of the bZIP dimer. These half-sites do not show any variation in the orientation of motifs, and show only a small change in spacer length from 1 to 4 bp [33]. In contrast, TFs from the NR class can function as monomers or dimers, and two half-sites can have a diverse structure with various orientations and spacers [32,33]. Therefore, a very important prerequisite to the analysis of homotypic CEs is the propensity of TFs to homotypic dimerization [45], i.e., dimerization among members of the same TF class. The review of Amoutzias et al. [45] indicated only three conservative TF classes with this ability common for *S. cerevisiae*, *H. sapiens,* and *A. thaliana* (fungi, animals, and plants, correspondingly). Namely, TFs from the bZIP, bHLH, and MADS box (MADS box factors {5.1}) classes emerged at the origin of eukaryotes. Whereas bZIP and bHLH TFs have undergone independent lineage-specific expansion in plants and animals, MADS-box TFs have done so only in plants; this class is very scarce in mammals [48]. Besides these three classes, the review [45] distinguished the following clades of TFs with homotypic dimerization propensity: the NR and STAT classes, and the families NF-κB and HD-ZIP. The NR class is specific to metazoa [49]. The class STAT emerged [50] early in metazoan evolution. The NF-κB family (NF-kappaB-related factors {6.1.1}) belongs to the class RHR; this family is also specific to metazoa. The plant-specific HD-ZIP family belongs [11] to the class Homeo domain factors {3.1}; this family emerged during the early chlorophyte evolution [51]. Taking into account the specific TF classes and families noted for their dimerization ability [45], we may conclude that among them, the bHLH class shows the most significant asymmetry in the conservation of motifs in homotypic CEs with a spacer (Figure 5, Figure 6 and Figures S1 and S3). The structurally related bZIP class shows similar results, but it is still superior to all other classes. The NF-κB family and the MADS-box class reveal a moderate significance of asymmetry, whereas the NR and STAT classes achieve a relatively low one. The conclusion about the superiority of the bZIP and bHLH classes holds for all tested thresholds of asymmetry (TAR) for the *M. musculus* ChIP-seq data and for the *A. thaliana* ChIP-seq/DAP-seq data (Figure 5, Figure 6 and Figures S1–S4). Notably, we detected the second rank of significance of homotypic CEs for the small class of p53 domain factors {6.3} that exists only in mammals (Figure 6A). In the murine benchmark collection (Tables S1 and S4), this class contains only one TF, TPR53; its human orthologue is TP53 (tumor protein p53). The regulatory protein p53 is a very well-known tumor suppressor preventing cancer formation in mammals. The minimal p53-binding site [52] is composed of two half-sites of RRRCWWGYYY (R = A/G, W = A/T, Y= T/C) followed by a variable spacer of 0–21 bp. It was experimentally shown that each p53 monomer unit binds to one quarter-site, resulting in all four DNA quarter-sites being occupied by one p53 tetramer [53]. Each half-site is divided into two quarter-sites of RRRCW; the two half-sites share the same quarter-site orientations, and the tetramer p53 can bind half-sites containing quarter-sites in all possible orientations (Direct, Inverted or Everted, see Figure 1A). Many genes have clusters of sites consisting of three or more half-sites. A recent analysis [54] revealed that the discrimination energy of 5′ half-sites of p53-responsive elements negatively correlated with the discrimination energy of 3′ half-sites. The pioneer TFs demonstrated a higher significance of asymmetry within homotypic CEs than other TFs (Figure 8 and Figures S5 and S6). However, particular pioneer TFs from different classes showed very significant asymmetry (Figure 9 and Figure S7), e.g., CEBPA and CEBPB (bZIP), ARNTL and CLOCK (bHLH), SPI1 (Tryp C), EBF1 (RHR), and TRP53 (p53 domain factors {6.3}).

Regarding the mechanism underlying the asymmetry of motif conservation within homotypic CEs, we indicate an analogy with the mechanism [55] of ternary complex formation from two TF subunits of the dimer and DNA. Either one of the two subunits initially interacts with DNA independently and then it recruits the second subunit, or the two subunits initially interact with each other and then a TF dimer interacts with DNA. These two options are referred to as monomer and dimer pathways [55]. In this study, the efficiency of these pathways for AP1 heterodimer formation from the subunits cFos and cJun was experimentally evaluated. Both subunits have the bZIP DBD. It was demonstrated that, although the dimerization of two subunits occurred rapidly in the absence of DNA, its rate was enhanced in the presence of DNA. Therefore, the monomer pathway was favored. The monomer pathway implied the sequential binding of subunits to DNA. The sequential binding was shown [56] for other bZIP TFs, and for TFs from other classes, e.g., NR [57,58] and bHLH [59]. The results of our study propose that the asymmetry in the conservation of motifs in homotypic CEs reflects a sequential mode of cooperative binding of TFs from certain classes. Since the cooperative action of TFs from the same class is deciphered by homotypic CEs, and we detected the strong depletion of symmetric CEs for all TF classes, we believe that the sequential mechanism of formation of multiprotein complexes on DNA is ubiquitous. The nucleotide context of homotypic CEs of TFs from the bZIP and bHLH classes indicates that they are more prone to sequential binding than TFs of other classes.

The propensity to recruit cooperating partner TFs is a well-known fundamental property of TFs as principal regulators of gene transcription. We suppose that the significant enrichment of asymmetric homotypic CEs for TFs of any class shows their ability to recruit collaborative TFs. Obviously, among the diversity of TFs, pioneer TFs are the first to demand this recruiting potential. The significant difference in asymmetry within homotypic CEs between pioneer TFs and TFs lacking pioneer function suggests that asymmetry within CEs signifies the ability of TFs to recruit their partners to contiguous regions of genomic DNA (Figure 7, Figure 8 and Figures S5–S7). Although the higher recruitment capacity of pioneer TFs seems obvious, trying to decipher it as a part of the genomic regulatory code is an open challenge.

Another explanation of the co-occurrence of stronger and weaker binding sites described the weaker ones as ‘traps’ [32], or ‘antennas’ [60]. These weaker sites operate as attractive intermediate elements, providing a traffic of target TF molecules towards sites of higher affinity. We recently combined the traditional motif model PWM, neglecting the dependencies of various positions in motifs, and the alternative motif models BaMM [61] and SiteGA [62], allowing such dependencies in a massive analysis of ChIP-seq data. BaMM adopted the framework of the PWM, adding to its total recognition score the additional contributions of several closest base pair positions [61]. The SiteGA model applied a genetic algorithm and optimized the discriminant function of frequencies of locally positioned dinucleotides [63]. We found that the PWM model was successful in its prediction of sites of high conservation, whereas both alternative models efficiently complemented the predictions of the PWM at the recognition thresholds of sites with low conservation [62]. Thus, we hope that the further application of alternative motif models may clarify the nucleotide context of weaker motifs from asymmetric homotypic CEs.

Yet another explanation of the co-occurrence of stronger and weaker binding sites proposes that these different sites belong to distinct but structurally similar TFs from the same class. The widespread dimerization [45] among members of the same TF classes, such as bHLH, bZIP, NR, MADS-box, and STAT, supports this hypothesis. We checked whether recognized homotypic CEs could represent TF heterodimers as follows. We overlapped ChIP-seq datasets for the target TFs from the same classes, considered the overlapped peaks, and compared the significance of asymmetry of the homotypic and heterotypic CEs. The results (Figure 9) confirmed that for both tested TF classes, bHLH and bZIP, the two co-occurring motifs of two distinct TFs from the same TF class partially explained the phenomenon of the asymmetry of motif conservation within homotypic CEs. Yet this observation cannot completely explain the overrepresented asymmetry for all homotypic CEs. First, dimerization ability is common for a restricted structural subset [45] of all TFs, but we found that this asymmetry is widespread for target TFs of all classes (Figure 5 and Figure 6). Second, the analysis of ChIP-seq and DAP-seq data revealed almost identical results (Figure 5 and Figure 6); given that DAP-seq data completely exclude the influence of partner TFs, the asymmetry of motif conservation within homotypic CEs largely reflects the affinity of these binding sites for the same target TFs.

## 4. Materials and Methods

### 4.1. Benchmark Collections of ChIP-Seq and DAP-Seq Data and Their Preliminary Filtration

We compiled the benchmark collections of ChIP-seq data for *M. musculus* and *A. thaliana* from the GTRD [41], and the benchmark collection of DAP-seq data for *A. thaliana* from the Plant Cistrome [4]. The murine collection included datasets that were prepared only for normal tissues/organs. Thus, we tried to maximize the presence of CEs functioning in mice in vivo. For each ChIP-seq dataset, we required an input control experiment to be present in the raw data processing [41]. Raw ChIP-seq and DAP-seq data were processed in the GTRD by the peak caller MACS2 [39,64]. For all collections, we used the foreground sets of 1000 top-scoring full-length peaks not exceeding 3000 bp. We ensured that the target proteins of the ChIP-seq experiments were TFs. To confirm murine TFs, we applied the list of 1639 human TFs [12]; the high homology between human and murine TFs enabled this criterion [8]. To confirm plant TFs, we applied the annotations from PlantRegMap [65] and TAIR [66]. We used the AME tool [42] to confirm the enrichment of known motifs of target TFs. Thus, we initially extracted 1610 ChIP-seq datasets for *M. musculus* and 121/511 ChIP-seq/DAP-seq datasets for *A. thaliana* (Tables S1–S3). We used the hierarchical classification [6,7,8,9,10,11] of target TFs considering the structure of their DBDs.

### 4.2. De Novo Motif Search and Final Filtration of ChIP-Seq/DAP-Seq Data

We used the realization STREME [40] of the traditional motif model PWM for the de novo motif search to define the enriched motifs of the target TFs. For the de novo motif search, we extracted background sequence sets from reference genomes, taking the A/T content of the foreground sequences into account and exactly preserving their lengths [67].

For each ChIP-seq dataset, we ensured the significant similarity (*p*-value < 0.001, TomTom tool) [43] of the enriched motifs of the first ranks to known motifs from JASPAR [10], CisBP [28], or Hocomoco [29] belonging to TFs from the families/classes of the target TFs. We removed from all collections datasets assigned to the superclass ‘Yet undefined DNA-binding domains {0}’ [7,8,11], as well as those that lacked class specification. All ChIP-seq datasets passed the MCOT application criterion [21]. The application rejected several DAP-seq datasets, most probably due to representing simple sequence repeats (ABI3VP1_tnt.VRN1_col_a and ARID_tnt.AT1G20910_col_a, ND_tnt.FRS9_col_a, ND_tnt.FRS9_colamp_a). The final filtration provided 1281 *M. musculus* ChIP-seq datasets and 80/495 *A. thaliana* ChIP-seq/DAP-seq datasets for 196/40/311 target TFs, correspondingly. Tables S4–S6 show counts of the datasets and target TFs for all classes for the three finally compiled benchmark collections.

### 4.3. Composite Element Analysis

MCOT applied four basic attributes to describe the CEs. First, homo-/heterotypic CEs represent pairs of motifs recognized with identical/distinct motif models. The next attributes show the mutual orientations of two sites, their mutual location with a spacer or an overlap, and their conservation [21,34,35] (Figure 1 and Figure 2A). Here, we considered only homotypic CEs with a spacer. The analysis of homotypic CEs with an overlap of motifs may be more complicated due to the possible full or partial self-complementarity of two motifs in a CE. E.g., full and partial overlap cases refer to the E-box motifs of CANNTG, representing TFs from the bHLH class, and the polyG motifs of TFs from the Three-zinc finger Krüppel-related factors {2.3.1} family from the C2H2 ZF {2.3} class, respectively [7]. We used output motifs from the de novo motif search tool STREME [40] as Anchor motifs in MCOT. For each motif, MCOT applied the recognition thresholds according to the preliminary computed table ‘Thr (recognition threshold) vs. –Log_10_(ERR) (expected recognition rate)’ for the whole-genome set of promoters of protein-coding genes [21,62]. For a given threshold, the *ERR* value is the ratio of the number of recognized sites in both DNA strands *N_REC_* to the number of total tested sites of promoters in all sequences, positions, and both DNA strands *N_TOT_*. The term ‘recognized’ means that predicted sites in promoters have the same or higher recognition score as the value in the ‘Threshold vs. ERR’ table.

$$ERR=\frac{N_{REC}}{N_{TOT}}$$

The maximal *ERR* is the parameter of MCOT; here, we accepted the value 0.001 for it [62]. The conservation C_i_ of each of two sites in a CE (i = 1, 2) is computed as follows: C_i_ = −Log_10_(ERR_i_). For a CE, MCOT computes the asymmetry ratio (AR) as the ratio between the largest and the smallest ERR values of a pair of sites (AR = Max (ERR_1_, ERR_2_)/Min (ERR_1_, ERR_2_)) to define whether this CE is asymmetric or symmetric (see Figure 2A). With a given input parameter, the threshold for the asymmetry ratio (TAR) is calculated either for homo- or heterotypic CEs. Asymmetric and symmetric CEs are defined as follows: if AR ≤ TAR, then a CE is symmetric; otherwise, if AR > TAR, then a CE is asymmetric.

To estimate the significance of enrichment to either asymmetric or symmetric CEs, MCOT performs the permutation procedure [21] (Figure 2B). It takes each foreground sequence (ChIP-seq peak) and generates many background sequences by separate random permutations of all clusters of overlapping site groups and the spacers between these groups. Further, total counts of the asymmetric and symmetric CEs in the foreground and background sequence sets are computed. Finally, the 2 × 2 contingency table (Figure 2C) provides the significance of the Fisher exact test, comparing the abundance [21,34] of CEs in the foreground and background sets. The sign ‘+’ in relation to asymmetry significance −Log_10_ [*p*-value] denotes [34] the enrichment of asymmetric CEs; otherwise, the sign ‘−’ denotes the enrichment of symmetric CEs. For an example, see Tables S7–S9.

The visualization of asymmetric/symmetric CEs was performed as described earlier [34] with small modifications. The options ‘Expected ERR’ and ‘Asymmetry ratio’ of MCOT [21,34,68] and its web-server WebMCOT [35,69] provided the threshold values of the maximal allowable ERR for any motif and the TAR value for any CE in the advanced options. We drew asymmetry heatmaps (Figure 4B) as follows [34]. For the foreground and background sets of sequences, we compiled the full lists of predicted CEs; for each CE, we computed the conservation of participant sites: {C_i_} = {−Log_10_(ERR_i_)}, i = 1, 2. The total counts of CEs for the foreground and background sets were denoted as Obs and Exp, respectively. The below indices j and k denote the range of the conservation of sites in the CEs. For instance, for the ERR value of 0.001, we define this range as follows: [<3.2], [3.2‥3.4], [3.4‥3.4], etc., up to [5.0‥5.2] and [>5.2] (see Figure 4B). We count CEs respecting all distinct combinations of the conservation of sites in the CEs for the foreground (Obs_j,k_) and background (Exp_j,k_) sets. Finally, we plotted the per mille measure, which converted the absolute CE counts to relative ones: [Relative Abundance] = {1000 × Obs_j,k_/Obs)} − {1000 × Exp_j,k_/Exp}.

### 4.4. Analysis of Pairs of ChIP-Seq Datasets

We analyzed the pairwise overlap of the ChIP-seq datasets as follows. We started from the benchmark collection for *M. musculus* (Table S4). We selected only pairs of datasets for distinct target TFs from the same classes. We considered separately the target TFs from the bHLH and bZIP classes. TFs from these classes act only as TF dimers [45]. Other classes either had too small numbers of dataset pairs (e.g., STAT, RHR), or their TFs may act as monomers (NR). For each pair of datasets, we required the same tissue/cell line/treatment conditions. We applied BEDtools [44] to confirm for each pair of datasets that at least 50 peaks from the first and the second datasets had at least 50% overlapped length, and the significance of the overlap (*p* < 0.001). We also ensured moderate similarity [43] between the enriched motifs identified by the de novo search from the first and second datasets (*p*-value < 0.05). Next, for each pair of datasets (for TF1 and TF2), we applied MCOT [21] to estimate the significance of the asymmetry within the CEs consisting of motifs of the same TF (homotypic CEs: TF1 vs. TF1, and TF2 vs. TF2) and within CEs consisting of motifs of distinct TFs (heterotypic CEs: TF1 vs. TF2).

## 5. Conclusions

We proposed a novel approach for the detection of pairs of co-occurring TFBS motifs specific to the asymmetry of their conservation in pairs. We considered only pairs of sites predicted with the same motif model (homotypic), and these pairs were located only with spacers. We prepared three benchmark collections for analysis: ChIP-seq data for *M. musculus* and *A. thaliana* from GTRD, and DAP-seq data for *A. thaliana* from Plant Cistrome. We used the TFClass and Plant-TFClass databases to categorize the target TFs from the ChIP-seq and DAP-seq experiments into classes, according to the structure of their DBDs. For all datasets, we performed a de novo motif search with the traditional PWM model. In the analysis, we only considered datasets that had enriched motifs of the corresponding target TFs. We considered homotypic pairs of motifs of target TFs and computed for each dataset the significance of the asymmetry/symmetry of motif conservation in these pairs. We demonstrated that major parts of all collections revealed significant asymmetry, while minor parts showed neither significant asymmetry nor symmetry. Only a few ChIP-seq datasets in the *M. musculus* collection showed significant symmetry. Among the large classes of TFs common to *M. musculus* and *A. thaliana,* three classes—bZIP {1.1}, bHLH {1.2}, and Tryptophan cluster factors {3.5}—showed a high significance of asymmetry. In *M. musculus*, two other classes—p53 domain {6.3} and Rel homology region {6.1}—also showed similarly high significance. We confirmed that target TFs with proven pioneer activity show more significant asymmetry within homotypic pairs of motifs compared to other TFs for which such activity is not known. Overall, our results argue that detecting trends of significant asymmetry within homotypic pairs of co-occurring motifs is a promising feature useful for figuring out mechanisms of gene transcription regulation.

## Data Availability

Data are contained within the article

## Supplementary Materials

The following supporting information can be downloaded at: https://www.mdpi.com/article/10.3390/ijms26010386/s1.

## Abbreviations

The following abbreviations are used in this manuscript:

AR	asymmetry ratio
bHLH	Basic helix–loop–helix factors
BS	binding site
bZIP	Basic leucine zipper factors
C2H2 ZF	C2H2 zinc finger factors
CE	composite element
DBD	DNA-binding domain
ERR	expected recognition rate
NR	Nuclear receptors with C4 zinc fingers
PWM	position weight matrix
RHR	Rel homology region factors
STAT	STAT domain factors
TAR	threshold for asymmetry ratio
TF	transcription factor
TFBS	transcription factor binding site
Tryp C	Tryptophan cluster factors
