# Supplementary material for: Asymmetry of Motif Conservation Within Their Homotypic Pairs Distinguishes DNA-Binding Domains of Target Transcription Factors in ChIP-Seq Data
Source: Int J Mol Sci. 2025 Jan 4;26(1):386. doi: 10.3390/ijms26010386 (PMC11720554; doi:10.3390/ijms26010386)
Supplement: Supplementary file 1 [file ijms-26-00386-s001.zip › Supp_Figures.pdf]

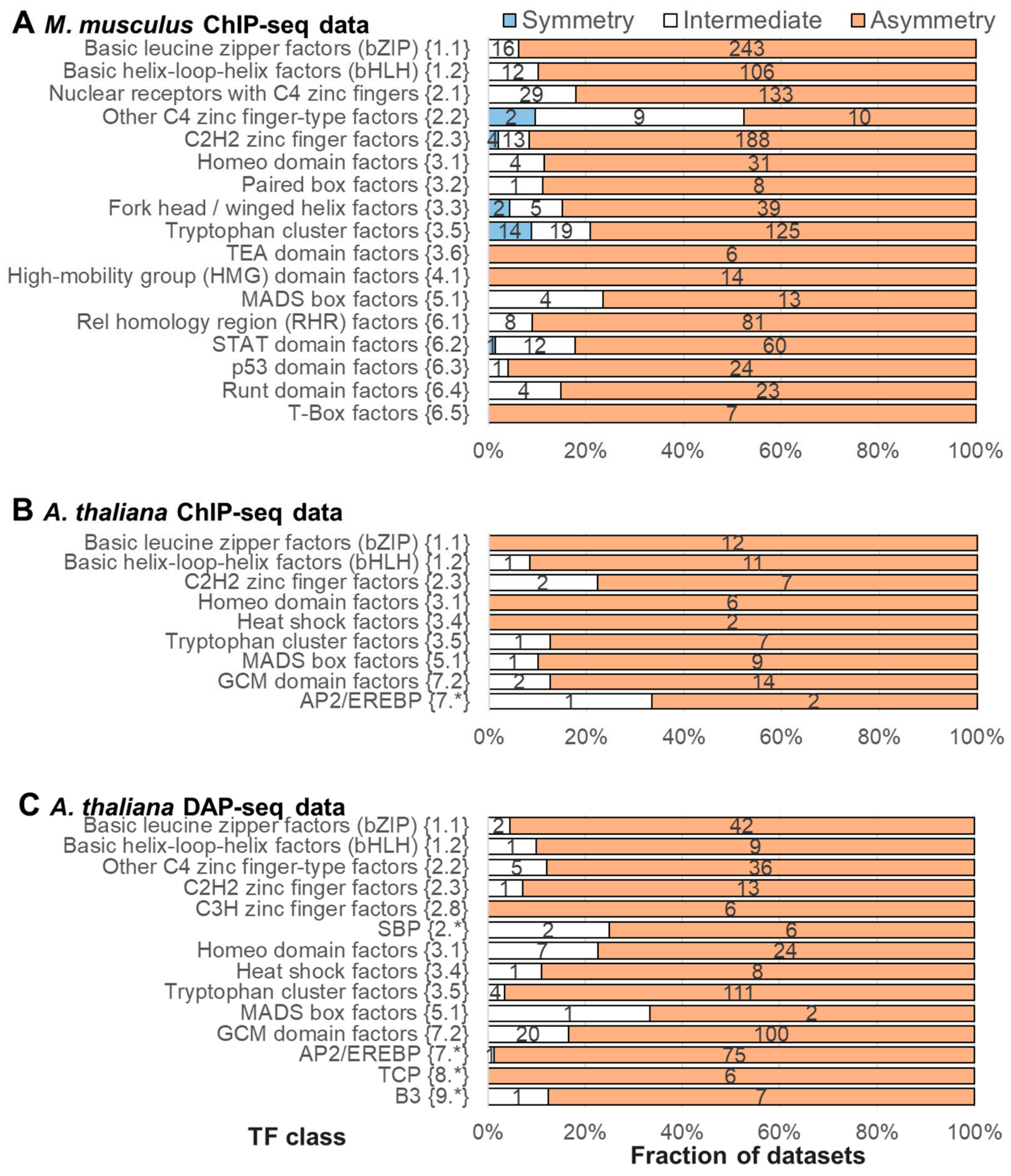

Figure S1. Abundances of hom
for TAR value of 1.1**.** (**A**) ChIP-se
seq data for *A. thaliana.* (**C**) DAF
show the number of datasets in a
datasets possessing a high s
homotypic symmetric/asymmetr
color respects absence of a h
neither symmetric nor asymme
Axes Y display TF classes acc
Plant-TFClass [11], (**B**) and (**C**).
A. thaliana ChIP-seq/ DAP-seq
and 2/3 datasets, corresponding

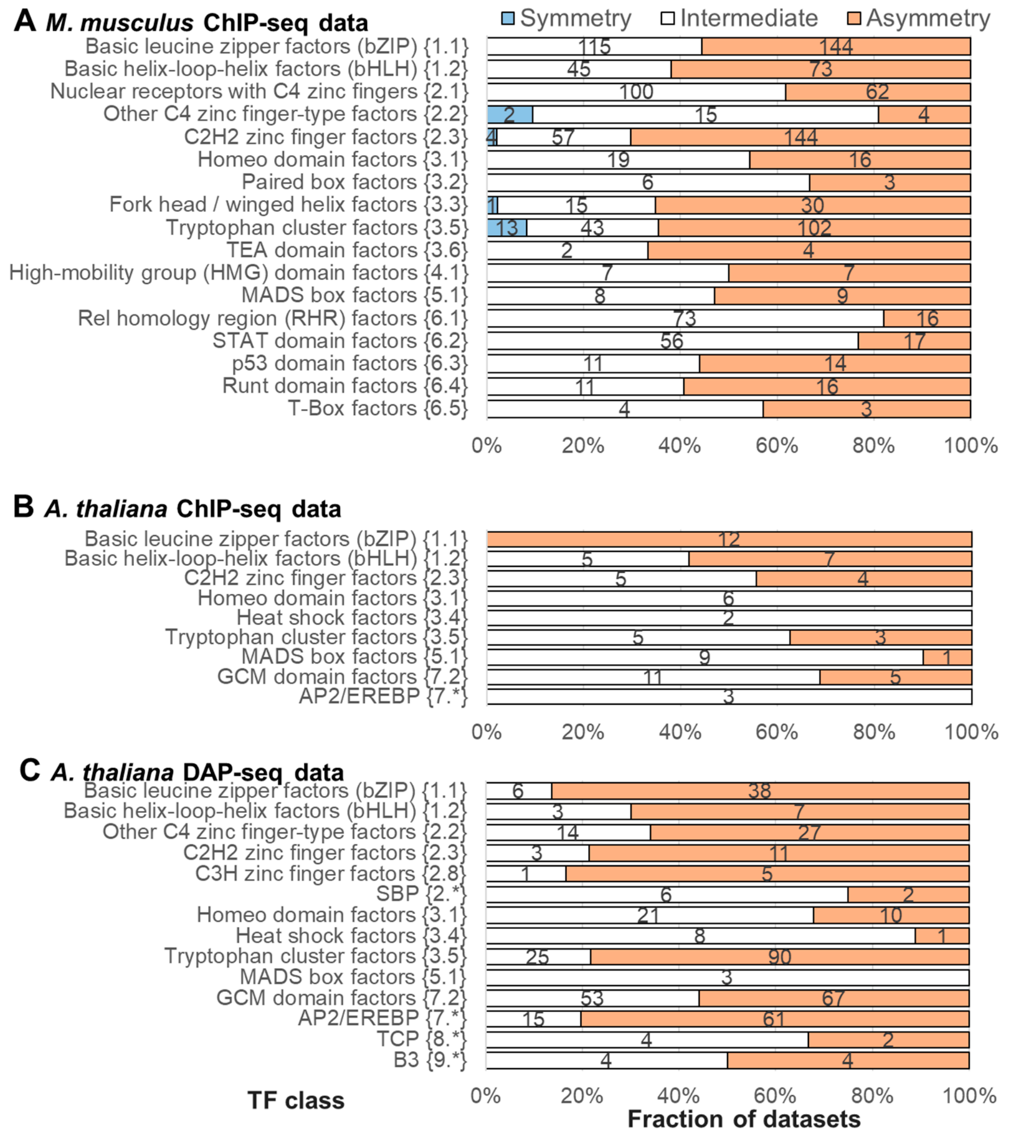

Figure S2. Abundances of homotypic asymmetric/symmetric CEs for TAR value of 2. (**A**) ChIP-seq data for *M. musculus.* (**B**) ChIP-seq data for *A. thaliana.* (**C**) DAP-seq data for *A. thaliana.* Axes X show the number of datasets in a TF class. Blue/brown colors count datasets possessing a high significance of enrichment within homotypic symmetric/asymmetric CEs, P-value < 10^{-10}. White color respects absence of a high significance, P-value > 10^{-10}, neither symmetric nor asymmetric CEs have high significance. Axes Y display TF classes according to TFClass [7,8] (A) and Plant-TFClass [11], (B) and (C). For *M. musculus* ChIP-seq data, *A. thaliana* ChIP-seq/ DAP-seq data, only classes with at least 6 and 2/3 datasets, correspondingly, are shown.

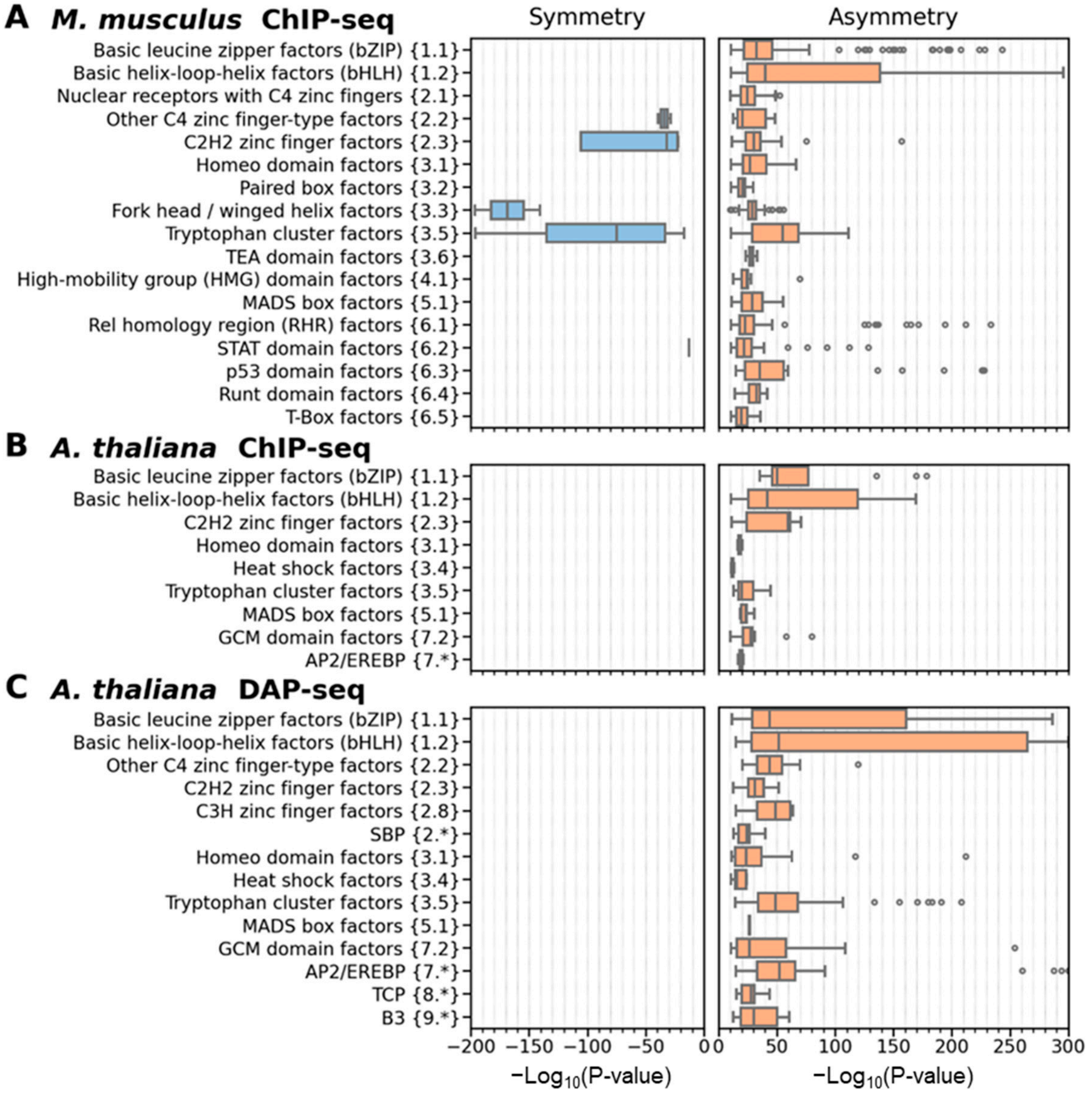

Figure S3. Distributions of the significance of enrichment of homotypic asymmetric/symmetric CEs as a function of the class target TF, TAR value of 1.1 is applied. (**A**) ChIP-seq data for *M. musculus.* (**B**/**C**) ChIP-seq/DAP-seq data for *A. thaliana.* Hierarchical classifications of target TFs by the structure of DBDs were derived from TFclass [7,8] and Plant-TFclass [11]. Axes X mark the significance of enrichment by Fisher exact test, $-\text{Log}_{10}$(P-value), calculated by MCOT [21]. Brown/blue colors imply enrichment towards asymmetry/symmetry, for each TF class we considered only datasets possessing the significant enrichment towards asymmetry or symmetry according to the results from Figure 5. Axis Y mark TF classes. The boxplots depict the distributions of the Q_1, Q_2 and Q_3 quartiles of the fractions of the considered datasets with certain values of the significance, $-\text{Log}_{10}$(P-value). Whiskers on either side of the Q_1/Q_3 respect the minimum/maximum values if they were located within 1.5 interquartile ranges (IQR = $Q_3 - Q_1$) from Q_1/Q_3, otherwise they are equal to {Q_1 - 1.5 * IQR} / {Q_3 + 1.5 * IQR}, respectively. In the latter case, we marked all other points as outliers.

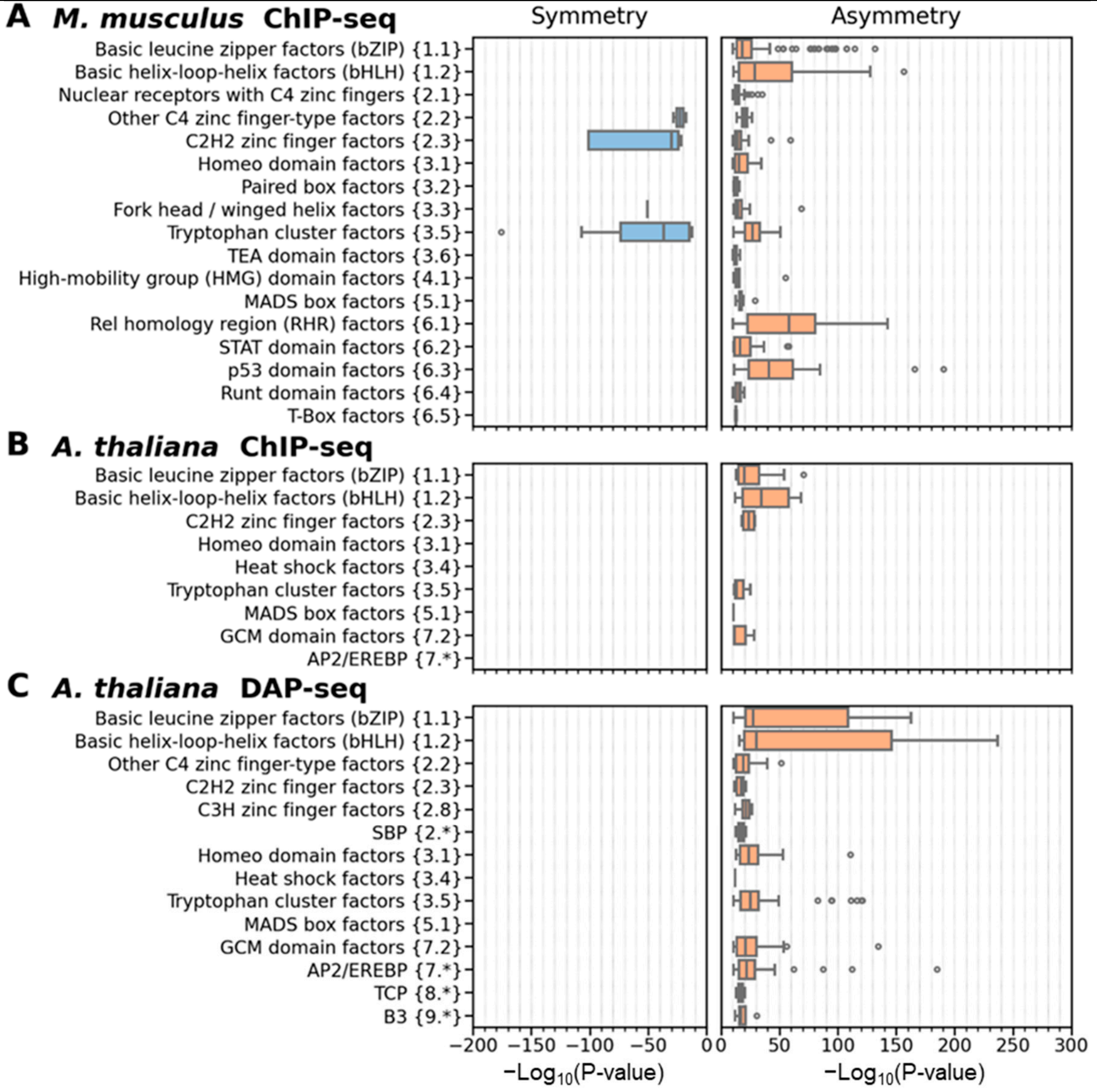

Figure S4. Distributions of the significance of enrichment of homotypic asymmetric/symmetric CEs as a function of the class target TF, TAR value of 2 is applied. (**A**) ChIP-seq data for *M. musculus.* (**B**/**C**) ChIP-seq/DAP-seq data for *A. thaliana.* Hierarchical classifications of target TFs by the structure of DBDs were derived from TFclass [7,8] and Plant-TFclass [11]. Axes X indicate the significance of enrichment by Fisher exact test, $-\text{Log}_{10}$(P-value), calculated by MCOT [21]. Brown/blue colors imply enrichment towards asymmetry/symmetry, for each TF class we considered only datasets possessing the significant enrichment towards asymmetry or symmetry according to the results from Figure 5. Axes Y mark TF classes. The boxplots depict the distributions of the Q_1, Q_2 and Q_3 quartiles of the fractions of the considered datasets with certain values of the significance, $-\text{Log}_{10}$(P-value). Whiskers on either side of the Q_1/Q_3 respect the minimum/maximum values if they were located within 1.5 interquartile ranges (IQR = $Q_3 - Q_1$) from Q_1/Q_3, otherwise they are equal to {Q_1 – 1.5 × IQR} / {Q_3 + 1.5 × IQR}, respectively. In the latter case, we marked all other points as outliers.

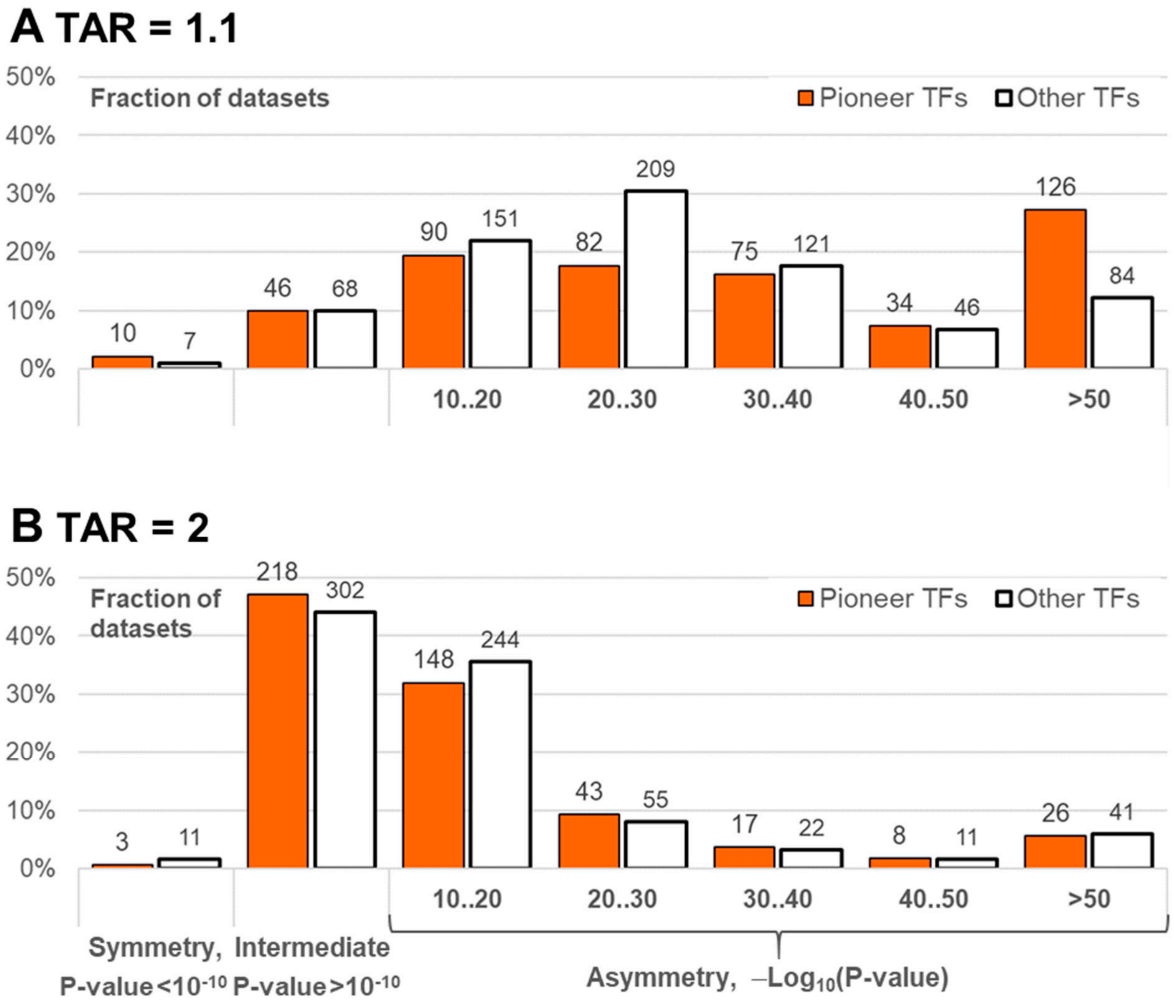

Figure S5. Distributions of the significance of enrichment of homotypic asymmetric CEs for ChIP-seq datasets of target TFs with and without proven pioneer activity. TAR values of 1.1 (**A**) and 2 (**B**) are applied. Target TFs from the benchmark collection for *M. musculus* ChIP-seq data were considered. Orange and white columns indicate TFs with and without proven pioneering activity. Axis X denotes the significance of enrichment. The groups Symmetry, Intermediate, Asymmetry 10..20, Asymmetry 20..30, etc. imply the significant enrichment of symmetric CEs (P-value < 10^{-10}), lack of high significance in either direction (P-value > 10^{-10}), the significant enrichment of asymmetric CEs (10^{-20} < P-value < 10^{-10}, 10^{-30} < P-value < 10^{-20}, etc.), respectively. Axis Y means fractions of datasets, labels above columns show the number of datasets.

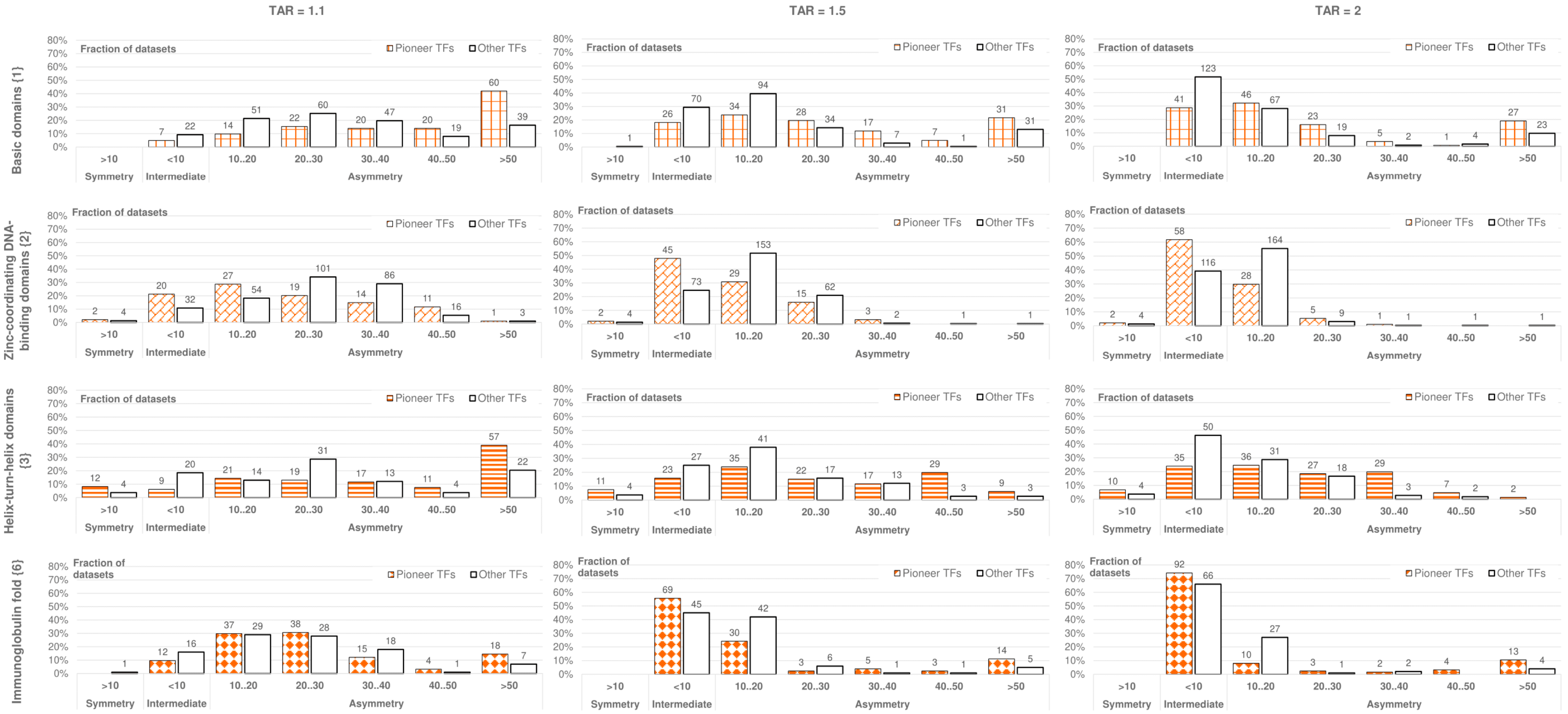

Figure S6. Homotypic asymmetric CEs of pioneer TFs from the four superclasses: Basic domains {1}, Zinc-coordinating DNA-binding domains {2}, Helix-turn-helix domains {3}, and Immunoglobulin fold {6} (rows). Target TFs from the benchmark collection for *M. musculus* ChIP-seq data were considered. TAR values of 1.1 (left), 1.5 (middle) and 2 (right) were applied to compute the significance of asymmetry within homotypic CEs. Four rows and different types of shading mark four superclasses (see labels at the left margin). Orange/white colors mark distributions for motifs of target TFs with/without proven pioneering activity. Distributions of the significance of asymmetry within CEs for ChIP-seq datasets of target TFs with and without proven pioneering activity. Axes X denote the significance of enrichment. The groups Symmetry, Intermediate, Asymmetry 10..20, Asymmetry 20..30, etc. imply the significant enrichment towards symmetric CEs (P-value < 10^{-10}), lack of pronounced significance in either direction (P-value > 10^{-10}), the significant enrichment towards asymmetric CEs (10^{-20} < P-value < 10^{-10}, 10^{-30} < P-value < 10^{-20}, etc.). Axes Y mean fractions of datasets, labels above columns show the numbers of datasets.

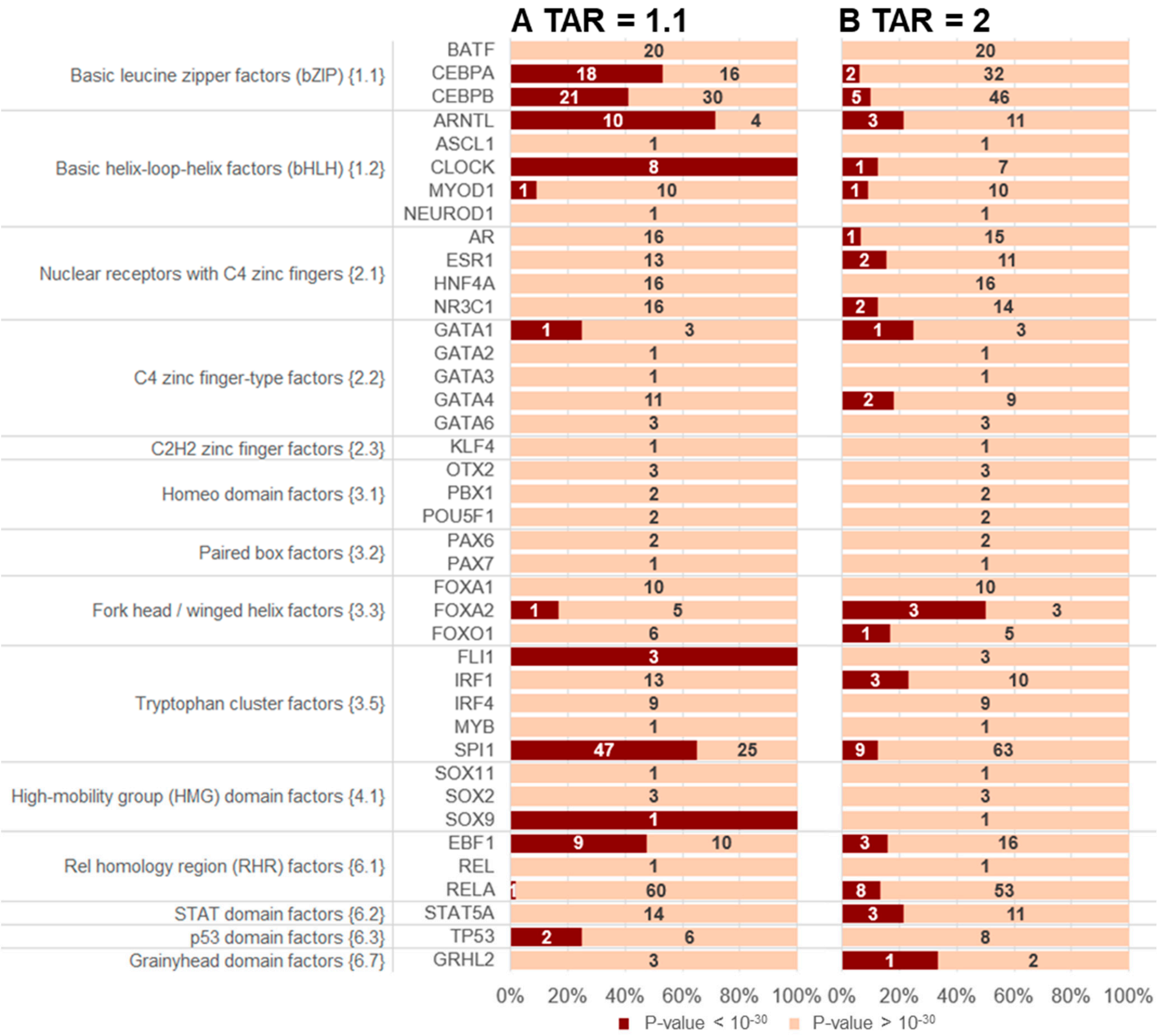

Figure S7. Pioneer target TFs with significant homotypic asymmetric CEs. TAR values of 1.1 (**A**) and 2 (**B**) were applied. Distributions of the significance of asymmetry within CEs for datasets from the benchmark collection of *M. musculus* ChIP-seq data for target TFs with proven pioneering activity.

Axis X marks the number of ChIP-seq datasets. Axis Y shows TF classes and TF names.

The maroon and apricot stripes indicate the high significance of asymmetry in homotypic CEs (P-value < 10^{-30}) and all remaining cases (P-value > 10^{-30}).
